# Sustainable Chitosan/Polybenzoxazine Films: Synergistically Improved Thermal, Mechanical, and Antimicrobial Properties

**DOI:** 10.3390/polym15041021

**Published:** 2023-02-17

**Authors:** Thirukumaran Periyasamy, Shakila Parveen Asrafali, Chaitany Jayprakash Raorane, Vinit Raj, Divya Shastri, Seong-Cheol Kim

**Affiliations:** 1 School of Chemical Engineering, Yeungnam University, Gyeongsan 38541, Republic of Korea; 2 School of Pharmacy, Yeungnam University, Gyeongsan 38541, Republic of Korea

**Keywords:** polybenzoxazine, biopolymer, ring-opening polymerization, antibacterial, antibiofilm activity

## Abstract

Polybenzoxazines (Pbzs) are considered as an advanced class of thermosetting phenolic resins as they overcome the shortcomings associated with novolac and resole type phenolic resins. Several advantages of these materials include curing without the use of catalysts, release of non-toxic by-products during curing, molecular design flexibility, near-zero shrinkage of the cured materials, low water absorption and so on. In spite of all these advantages, the brittleness of Pbz is a knotty problem that could be solved by blending with other polymers. Chitosan (Ch), has been extensively investigated in this context, but its thermal and mechanical properties rule out its practical applications. The purpose of this work is to fabricate an entirely bio-based Pbz films by blending chitosan with benzoxazine (Bzo), which is synthesized from curcumin and furfuryl amine (curcumin-furfurylamine-based Bzo, C-fu), by making use of a benign Schiff base chemistry. FT-IR and ^1^H-NMR spectroscopy were used to confirm the structure of C-fu. The impact of chitosan on benzoxazine polymerization was examined using FT-IR and DSC analyses. Further evidence for synergistic interactions was provided by DSC, SEM, TGA, and tensile testing. By incorporating C-fu into Ch, Ch-grafted-poly(C-fu) films were obtained with enhanced chemical resistance and tensile strength. The bio-based polymer films produced inhibited the growth of *Staphylococcus aureus* and *Escherichia coli*, by reversible labile linkages, expanding Ch galleries, and releasing phenolic species, which was 125 times stronger than bare Ch. In addition, synthesized polybenzoxazine films [Ch/Poly(C-fu)] showed significant dose-dependent antibiofilm activity against *S. aureus* and *E. coli* as determined by confirmed by confocal laser scanning microscopy (CLSM). This study suggests that bio-based Ch-graft-polymer material provide improved anti-bacterial property and characteristics that may be considered as a possibility in the near future for wound healing and implant applications.

## 1. Introduction

Biocompatibility, biodegradability, and sustainability are advantages of natural polymers over synthetic polymers. However, applications of natural polymers are limited by their poor mechanical and thermal properties, despite their benefits. Synthetic polymers can be combined with biopolymers which can mitigate these disadvantages. Polymer blends and copolymers may possess enhanced properties and be employed for a variety of applications as a result of the hybridization of biomacromolecules and synthetic polymers [1,2,3,4,5]. Recently, research institutes, public institutions, academia, and industry have paid increasing attention to polymer materials that are naturally biodegradable and biocompatible. Biomacromolecules such as chitosan (Ch) have been extensively studied and used over the last few decades [6,7,8,9]. Ch is a modified, high-molecular-weight, branched polysaccharide that is derived from shells of crabs and shrimps by deacetylation. In terms of available biomacromolecules, chitin ranks second worldwide. The unique physicochemical properties of chitosan-based biopolymers are due to the presence of reactive hydroxyl (–OH) and amine (–NH_2_) groups that can easily combine with other polymers to form blends [10,11,12,13,14]. Chitosan can be dissolved in dilute acids, such as citric, acetic, propionic, lactic acids, but is insoluble in water and various organic solvents. Ch has been extensively used in wastewater treatment, food, agriculture, and medicine because of its low cost, environmental friendliness, and high chemical reactivity. The disadvantage of Ch is that it is brittle and has poor thermo-mechanical performance. Ch chains are commonly grafted or crosslinked with other polymers, such as cellulose, starch, polyamide, genipin, acrylamide, polyaniline, aldehydes, or polyamide to mitigate these weaknesses [15,16,17,18,19,20,21,22,23,24,25,26,27].

Recently, polybenzoxazines have been developed as a class of thermoset polymers. As well as being mechanically strong, thermally stable, flame retardant, chemically and electrically resistant, and exhibiting virtually no shrinkage during polymerization process, these polymers also exhibit unique properties that distinguish them from conventional polymers, such as their reactivities with other polymers and their low dielectric properties. Holly and Cope first prepared benzoxazine monomers in 1944 by combining a phenol primary amine and formaldehyde [28]. According to Burke, Benzoxazine rings form Mannich bridges when attached to free ortho-positions of phenolic compounds. In order to obtain polybenzoxazines, benzoxazine monomers are converted into polybenzoxazines by thermally activating ring-opening polymerization without a catalyst or initiator being added [29,30,31], and notably no hazardous materials, toxic gases, or by-products are produced during the polymerization process. Using Mannich condensation formaldehyde, phenol, and an amine, benzoxazine monomers are synthesized. Recently, the use of fully biobased compounds has been actively studied. These bio-based resins combine the dimensional stability and chemical resistance of thermoplastics with the ease of processing and film-forming properties of thermosetting resins. Many polybenzoxazine alloys, blends, and composites have been developed to overcome the deficiencies of neat benzoxazine materials [32,33,34], and the addition of benzoxazine groups to the copolymers, they exhibit improved properties. The production of benzoxazines from renewable organic materials has recently received considerable attention. Some benzoxazine monomers are soluble in water and aqueous solutions, which are also eco-friendly solvents. A study by Omura et al. [35] reported that benzoxazine could functionalize Ch. In this study, Ch was blended with synthesized benzoxazine. The ring-opening polymerization of benzoxazine was used to evaluate the properties of mixtures containing Ch and benzoxazine. An aqueous solution containing a blend of Ch/polybenzoxazine is investigated in this study. We intend to quantify the effect of the addition of benzoxazine moiety to Ch in aqueous medium on mechanical and thermal properties, utilizing both their crosslinking capabilities and their attractive attributes.

## 2. Materials and Methods

### 2.1. Chemicals and Materials

Curcumin, furfuryl amine, chitosan, and paraformaldehyde were purchased from Sigma-Aldrich (St. Louis, MO, USA). Chloroform, acetic acid, and sodium hydroxide (NaOH) were purchased from Duksan Chemicals Co., Ltd., Incheon, Republic of Korea. Ethanol was purchased from Daejung Chemicals Co., Ltd., Gyeonggi-do, Republic of Korea.

### 2.2. Synthesis of Curcumin-Furfuryl Amine Based Benzoxazine Monomer (C-fu)

**Scheme 1:**
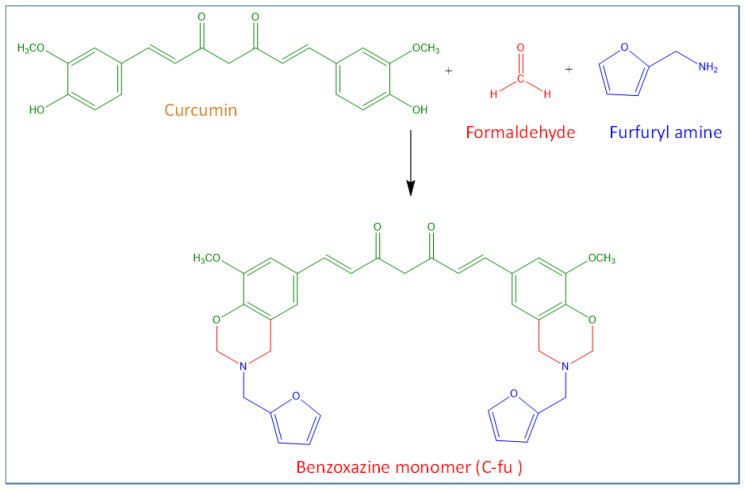
Synthesis of benzoxazine monomer (C-fu).

**Scheme 2:**
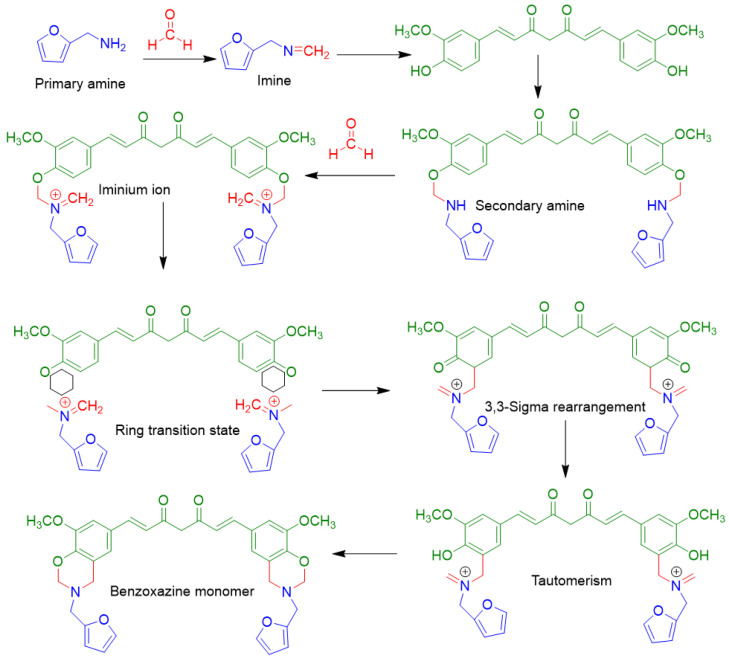
Mechanism of benzoxazine monomer synthesis.

The benzoxazine monomer based on curcumin and furfuryl amine, hereafter abbreviated as C-fu, was synthesized using the method described by Thirukumaran et al. [36] and shown in Scheme 1. In a 250 mL round bottomed flask, 80 mL of anhydrous chloroform, 0.01 mol of curcumin, 0.04 mol of paraformaldehyde, 0.02 mol of furfuryl amine, and 0.25 mol of CaH_2_ were taken. The reaction mixture was stirred for 6 h at 120 °C. After completion of the reaction, it was cooled to room temperature, and the product was filtered. The filtrate was evaporated under reduced pressure to remove the residual solvent. The evaporated solution was poured into excess of methanol to precipitate the product. The product was agitated for 30 min, filtered, washed several times with DI water and dried at 60 °C for 24 h under vacuum to obtain C-fu with 80% yield. Mechanism for the formation of benzoxazine monomer is shown in Scheme 2.

### 2.3. Synthesis of Ch/C-fu Blends

We prepared Ch/C-fu blends of different weight ratios: (100/0), (80/20), (60/40), (40/60), (20/80), and (0/100), which we referred to as 0, 20, 40, 60, 80, and 100% of C-fu blends respectively. Blends were prepared by diluting 30 mL of 1 wt% acetic acid solution with the required amount of Ch and benzoxazine monomer until a solid content of 2% by wt was achieved. Briefly, Ch was dissolved by continuous magnetic stirring in a solution of 1 wt% acetic acid at RT. While the Ch solution was being stirred, a specified amount of C-fu was dissolved in 1 wt% acetic acid solution and vigorously agitated using a magnetic stirrer to achieve a homogeneous mixture. The C-fu solution was then gradually added to the Ch solution. Then, the mixture was kept under vacuum at room temperature to remove any air bubbles present in it. After removing the air bubbles, the solution was poured onto a glass Petri dish and allowed to dry for 2 days at room temperature to form a uniform thick film. Afterwards, the films were dried at 50 °C for 24 h in a vacuum oven, where free standing films were obtained. Then, they were heated sequentially in an air-circulating oven for 2 h at 100, 125, 150, 175, and 200 °C to complete the cross-linking process. Film colors varied from light yellow to dark brown according to the amount of polybenzoxazine present. Cured films were denoted as poly(Ch/C-fu) (100/0); poly(Ch/C-fu) (80/20); poly(Ch/C-fu) (60/40); poly(Ch/C-fu) (40/60); poly(Ch/C-fu) (20/80); and poly(Ch/C-fu) (0/100).

### 2.4. Instrumentation Methods

The synthesized materials were thoroughly characterized by various physicochemical techniques. Fourier transform infrared (FT-IR) spectra were obtained with a Perkin Elmer MB3000 FTIR spectrometer (Waltham, MA, USA). The spectra were obtained at a resolution of 4 cm^−1^ in the IR range of 400–4000 cm^−1^. Samples were prepared by grinding with KBr and compressed to form discs. Nuclear magnetic resonance (NMR) spectrum was recorded by using an Agilent NMR, VNS600 at a proton frequency of 600 MHz for ^1^H-NMR. Solution was prepared by dissolving the sample in DMSO-d_6_. Differential scanning calorimetry was performed in a TA instrument Q10 model using 5–10 mg of the sample at a heating rate of 10 °C min^−1^ in a N_2_ atmosphere. Mechanical properties of cross-linked films were evaluated under uniaxial tension in an Instron E300LT, Universal Tester (Waltham, MA, USA). Five specimens for each composition were tested at ambient temperature using 1 mm/min as cross-head speed and the average value was reported. The reported modulus values were calculated from the slope of the stress–strain curve at 1% strain. Thermogravimetric analysis (TGA) was performed using a TA Q600 thermal analyzer, (New Castle, DE, USA). Cured samples were analyzed in an open silicon pan at a heating rate of 20 °C min^−1^ in a N_2_ atmosphere, up to a maximum temperature of 800 °C. Field emission scanning electron microscopy (FESEM) images were observed on a Hitachi S-4800, (Urbana, IL, USA) equipped at an accelerating voltage of 4 kV.

### 2.5. In Vitro Antibacterial Activity and MIC Determination

The antibacterial activity of poly(Ch/C-fu) films was tested in vitro using the disk diffusion method [37,38]. For this study, one Gram-negative bacteria *Escherichia coli* (*E. Coli*) ATCC 43895 and one Gram-positive bacteria *Staphylococcus aureus* (*S. aureus*) ATCC 6538 were used. Overnight cultures of each strain at 0.5 McFarland standard were seeded onto sterile Mueller Hinton agar (MHA) plates using sterile cotton swabs. The poly(Ch/C-fu) films discs (7-mm diameter) were impregnated with 20 μL test compound (with a concentration of 10 mg/mL); surface sterilization of discs was carried out using 100% ethanol. After incubation at 37 °C for 24 h, radii of inhibition zones were measured with a Vernier caliper. MIC was defined as the lowest concentration that inhibited cell growth in accordance with the Clinical Laboratory Standards Institute (CLSI) guideline [39], and assays were performed by adding freshly grown cells to cation-adjusted Mueller Hinton broth. Experiments were performed using at least three independent cultures.

### 2.6. Antibiofilm Potency of Ch/Poly (C-fu) (40/60) against E. coli and S. aureus

Biofilm assays were performed by crystal violet staining in 96-well microtiter plates, as previously described [40]. The initial turbidity of OD 0.05 (~10^6^ CFU mL^−1^) for *S. aureus* and OD 0.1 (~10^6^ CFU mL^−1^) for *E. coli* at 600 nm were inoculated into a LB culture media (final volume 300 μL) with Ch/Poly (C-fu) (40/60) at concentration of 0, 5, 10, 25 μg/mL and incubated for 24 h without shaking at 37 °C, and biofilm formation was confirmed by staining with 0.1% crystal violet for 30 min and washed frequently with distilled water and then 95% ethanol was added to each well. Absorbances were measured at 570 nm using a Spectramax 190 microplate reader equipped with a xenon flash lamp (Molecular Devices, San Jose, CA, USA). Biofilm assays were conducted twice independently in triplicate.

### 2.7. Biofilm Observations by Confocal Laser Scanning Microscopy

For the CLSM assay, single strain biofilms of *S. aureus* ATCC 6538 and *E. coli* ATCC 43895 were produced in 96-well plates with or without Ch/Poly (C-fu) (40/60) at 37 °C for 24 h without shaking [41]. Free-floating cells were then discarded by rinsing with water three times, and biofilm cells attached to the surface of the wells were stained with CFDA-SE (carboxyfluorescein diacetate succinimidyl ester) (Invitrogen, Molecular Probes, Inc, Eugene, OR, USA). The bottom of each well was then visualized using a 488 nm Ar laser (emission 500 to 550 nm) using a CLSM (Nikon, Tokyo, Japan). COMSTAT software was employed to determine the mean biofilm thicknesses (μm), biomass (μm^3^/μm^2^), and substratum coverages (%) and roughness coefficient [42]. Two independent samples were analyzed per experiment, and more than 12 random spots were observed.

## 3. Results and Discussion

### 3.1. Structural Confirmation of the Synthesized Benzoxazine Monomer (C-fu)

**Figure 1:**
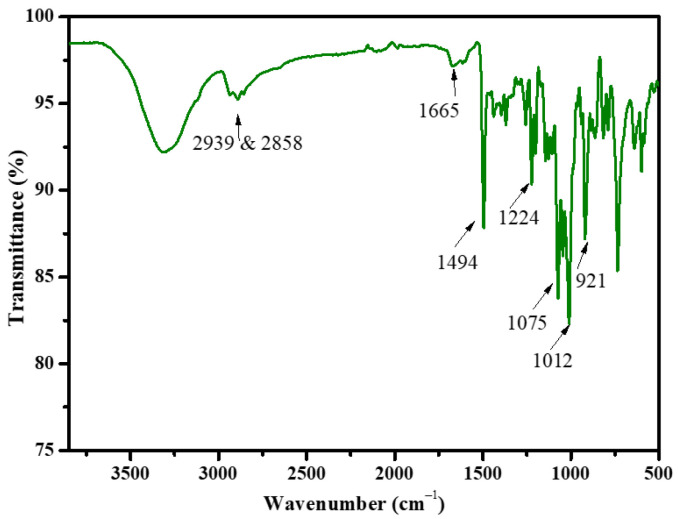
FT-IR spectrum of C-fu benzoxazine.

The FT-IR spectrum of benzoxazine monomer is shown in Figure 1. Asymmetric and symmetric stretching modes of C–O–C resulted in characteristic absorptions at 1232 cm^−1^ and 1024 cm^−1^ of the benzoxazine ring structure of C-fu, respectively. The peak at 934 cm^−1^ indicated the presence of an oxazine ring attached to a benzene ring. C–N–C symmetric stretching vibrations were also observed at 1146 cm^−1^. In addition, asymmetric and symmetric stretching vibrations of methoxy carbonyl attached to the benzene ring were observed at 1224 and 1012 cm^−1^, respectively. A band centered at 1654 cm^−1^ was attributed to C=O stretch of the curcumin moiety, and peaks at 1586, 994, and 731 cm^−1^ to vibrations of the furan ring. The C–H stretching modes at 2845 cm^−1^ and at 2954 cm^−1^ were attributed to symmetric and anti-symmetric stretching of alkyl chains and CH_2_ units of oxazine rings [43,44].

**Figure 2:**
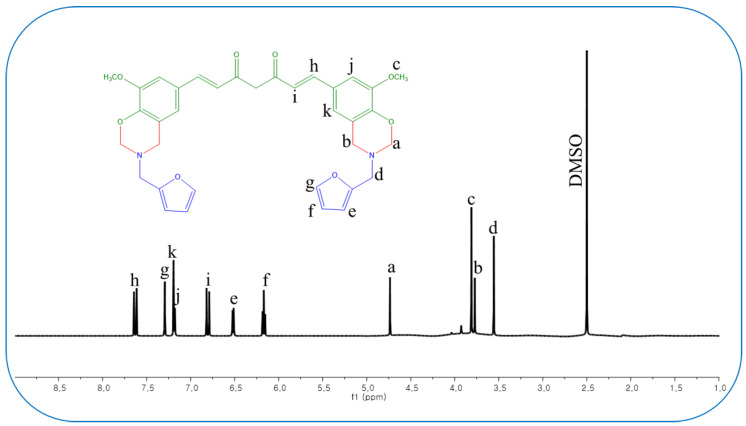
^1^H-NMR spectrum of C-fu benzoxazine.

^1^H-NMR spectrum is shown in Figure 2. Singlets at 3.9 and 4.7 ppm represent the classic protons associated with the oxazine ring, Ar–CH_2_–N and O–CH_2_–N, respectively. Doublets at 6.8 and 7.2 ppm, were attributed to the aromatic methine of curcumin, and a singlet at 3.75 ppm to the –OCH_3_ protons of curcumin, and a singlet at 3.75 ppm to the –OCH_3_ protons of curcumin. Another singlet at 3.75 ppm is due to the –OCH_3_ protons from curcumin moiety. The furan ring protons resonate at 6.2, 6.6, and 7.3 ppm, and multiplets between 6.5 and 7.3 ppm were assigned to aromatic protons. These results confirmed the formation of C-fu [45].

### 3.2. Polymerization Behavior of Poly(Ch/C-fu) Blends

**Scheme 3:**
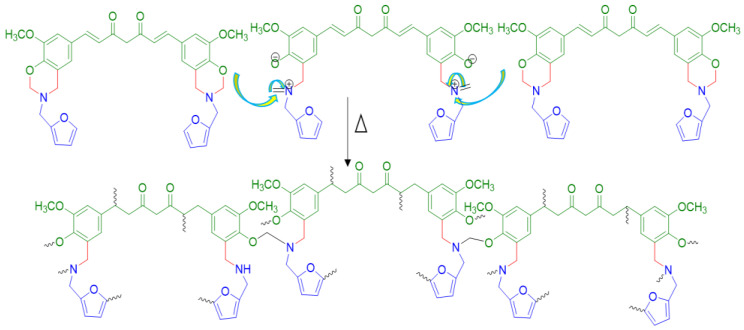
Thermally induced ring-opening polymerization of benzoxazine.

**Figure 3:**
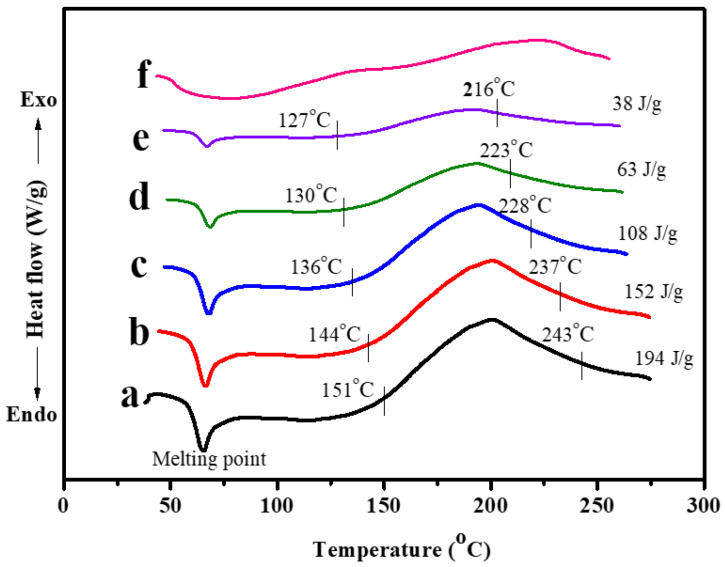
DSC thermograms for Ch/C-fu blends with different C-fu contents: (**a**) 100%, (**b**) 80%, (**c**) 60%, (**d**) 40%, (**e**) 20%, and (**f**) 0%.

**Scheme 4:**
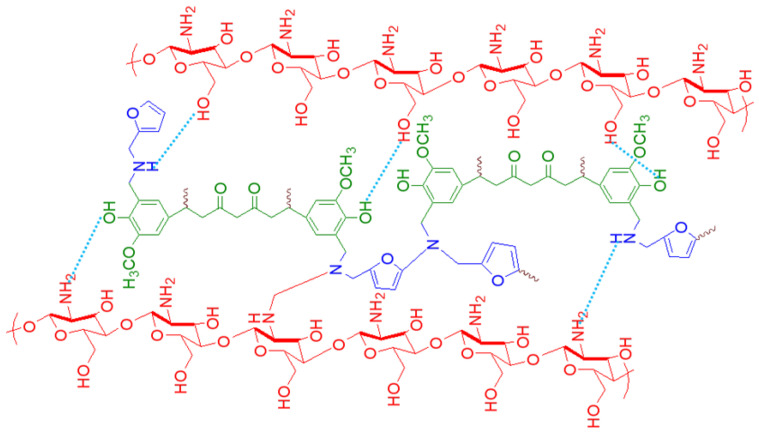
Representative co-polymerization mechanism Ch and C-fu.

Generally, benzoxazines undergo ring-opening polymerization via the formation of a Mannich base bridge (Scheme 3). C-fu underwent polymerization between 150 and 250 °C, as indicated by an exothermic peak. DSC thermograms of C-fu after curing at various benzoxazine weight ratios are shown in Figure 3. When C-fu was subjected to DSC, typical exothermic benzoxazine peak can be observed, which starts at 151 °C and ends at 243 °C. The amount of heat liberated, referred as enthalpy (∆H) during ring-opening polymerization for typical benzoxazine monomer is found between 100 and 250 J/g. DSC study at different Ch to C-fu weight ratios was conducted to determine whether Ch affects the benzoxazine polymerization or not. Comparing the Ch/C-fu blends to the neat benzoxazine, their exothermic peaks are entirely at a lower temperature (Table 1). Specifically, for Ch/C-fu (40/60), the onset of exotherm and final exotherm decreased from 151 to 136 °C and from 243 to 228 °C, respectively. Therefore, we can arrive at a conclusion that benzoxazine’s ring-opening polymerization is in part catalyzed by reactive amine groups of chitosan. Similar decrease in exothermic peak was observed with benzoxazine containing benzimidazole group as reported by Yang and Gu [46]. It can be viewed in such a way that the N–H group of the benzimidazole moiety, being a part of the network forms iminum ions by protonating the O atoms of benzoxazine rings. A reduction in the polymerization temperature of benzoxazine monomer is essential to inhibit certain difficulties including partial degradation. The polymerization of benzoxazine is supposed to be designated by a multifaceted catalytic interaction between OH and NH_2_/NH groups of chitosan and Pbz. This generally produces product with high T_g_ due to the formation of hydrogen bonding interaction. Scheme 4 represents the copolymerization mechanism in poly(Ch/C-fu). This synergistic effect may be explained in such a way that there are innumerable hydrogen bonds that are formed and competing with each other in the polymer structure. Several inter- and intramolecular hydrogen bonding interactions are formed between –NH group (in the Mannich base), –OH group of Pbz, and –NH_2_ and –OH group of chitosan. More detailed research is needed to understand this catalytic activity and hydrogen bond formation [47,48].

### 3.3. Tensile Properties of Poly(Ch/C-fu) Films

**Figure 4:**
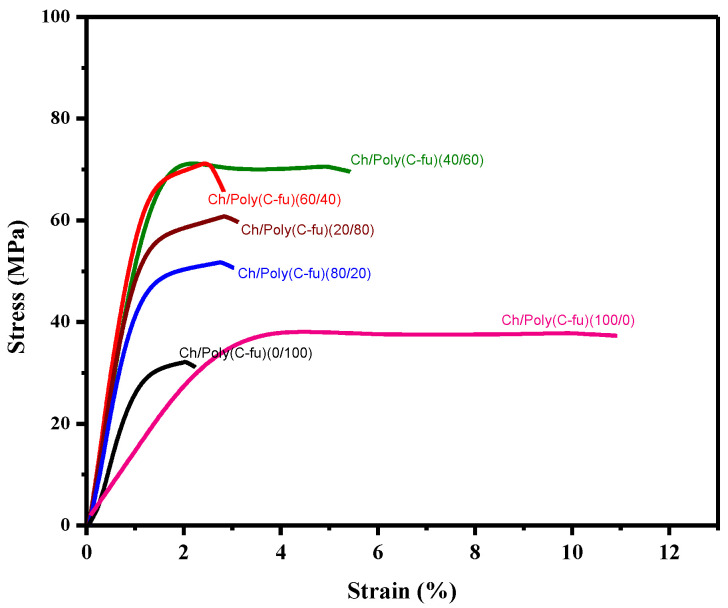
Stress–strain curves of poly (Ch/C-fu) films.

The tensile properties of poly(Ch/C-fu) films were examined and their stress–strain curves are presented in Figure 4. Notably, the molecular weight of Ch, the preparation medium, and the concentration of acid in the aqueous solution strongly influence the mechanical properties of Ch films. Following a maximum tensile strength of 35.3 MPa, the neat Ch film shows characteristic stretching performance to achieve an elongation at break value of 11%. Thermal treatment enhances the plasticity, and thus, reduces the brittleness of Ch films. As the benzoxazine content was increased in Ch/C-fu crosslinked polymers, tensile strength increased, but elongation at break decreased due to an increase in crosslink density (CLD). Poly(benzoxazine-*co*-urethane)s have shown similar results in previous reports [49]. Moreover, this CLD seems to be the reason for Bzo homopolymer’s crack sensitivity. When the C-fu content in the blend reached 60%, the crack tip is dampened and the potential strength of the benzoxazine polymer is fully realized. The results demonstrate that Ch and polybenzoxazine have an unusual synergistic effect, which enhances the strength of their co-polymer films. The tensile strength of Ch film increased from 35.3 MP to 75.2 MP when cross-linked with 60 wt% C-fu. It has been reported that physicochemical cross-linking of Ch with a second polymer has a synergistic effect on their mechanical properties. When Ch is blended with other polymers, it was found that the degree of deacetylation and average molecular weight are the main factors affecting the properties of cross-linked films.

### 3.4. Thermal Stabilities of Poly(Ch/C-fu) Films

**Figure 5:**
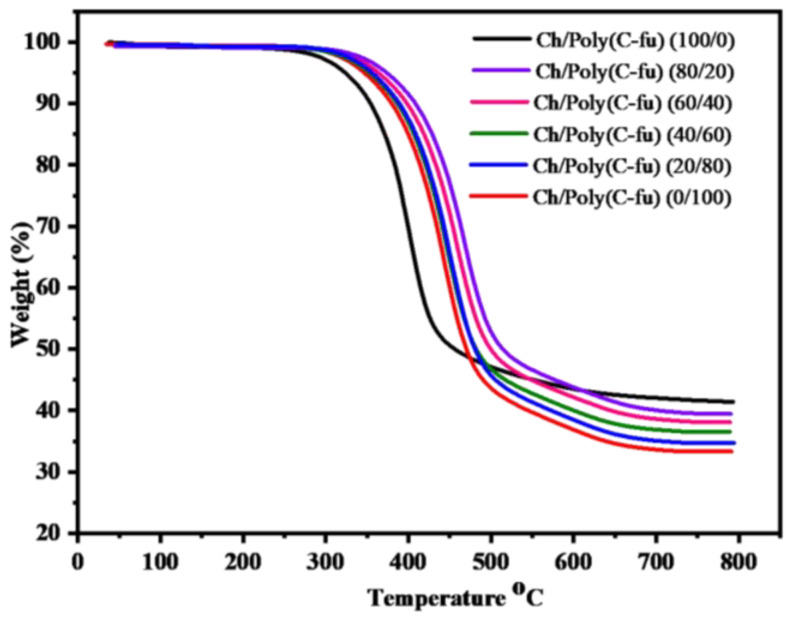
TGA thermograms of poly (Ch/C-fu) films.

The thermal stabilities of poly(Ch/C-fu) films were investigated by TGA under N_2_ (Figure 5). TGA thermograms showed maximum weight loss (about 50%) occurred between 200 and 450 °C, presumably due to the degradation of the chitosan moiety, which has a high aliphatic content. It is also observed that the neat Pbz, i.e., poly(C-fu) and its blends, i.e., poly (Ch/C-fu) have higher thermal stability compared to the neat chitosan. The decomposition temperature of Ch/poly(C-fu) films increased when compared with neat chitosan and neat poly(C-fu). This is due to the fact that amine moieties are directly incorporated into chitosan through cross-linking. Whereas neat chitosan film has the highest char yield of 43% at 800 °C. The neat Pbz and poly(Ch/C-fu) films have char yields between 34 and 40%. A significant synergistic effect results in considerable enhancement in thermal stability and char yield differences between the neat polymer and the cross-linked polymer [50,51,52,53]. Thermal properties were improved to a greater extent with the addition of 60% benzoxazine to cross-linked polymers. The 10% degradation temperature (T_10_) and maximum degradation temperature (T_50_) of poly(Ch/C-fu) films increased significantly with respect to the neat chitosan film from 350 to 410 °C and 450 to 520 °C, respectively. In addition, benzoxazine over 60 wt% did not show any significant increase in char yields.

### 3.5. Flame Retardant Properties of Poly(Ch/C-st) Films

Poly(Ch/C-fu) films were analyzed for flame retardancy using limiting oxygen index (*LOI*) values, which were calculated using the van Krevelan and Hofytzer equation [54] based on their char yield (*CY*) values from TGA analysis:

*LOI* = 17.5 + 0.4 (*CY*) (1)

For all the films, i.e., neat chitosan, neat Pbz, and poly(Ch/C-fu) the *LOI* value exceeded the threshold value of 26, and thus, all films were considered to possess self-extinguishing and flame retardant properties [55]. Accordingly, poly(C-fu) and poly(Ch/C-fu) were found to exhibit excellent mechanical, thermal, and flame-retardant properties.

### 3.6. Determination of the Antibacterial Efficacy of Poly(Ch/C-fu) Films

**Figure 6:**
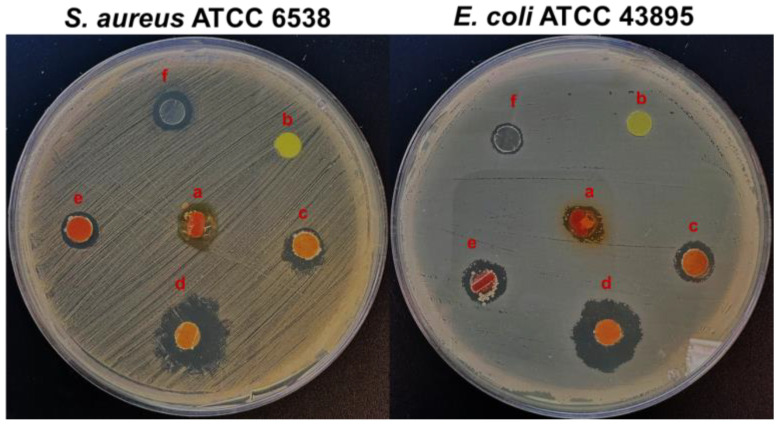
Antimicrobial activities of Ch/Poly (C-fu) blends with different C-fu contents: (a) 100%, (b) 80%, (c) 60%, (d) 40%, (e) 20%, and (f) 0%. Visible clear zones were produced by polybenzoxazine films against *S. aureus* and *E. coli*.

The Gram-positive and negative bacteria tests showed that both *S. aureus* ATCC 6538 and *E. coli* ATCC 43895 were susceptible to Ch/poly(C-fu) films. Disk diffusion tests showed Ch/poly(C-fu) films produced a clear zone against both strains. The diameters of inhibition zones are summarized in Table 2 and illustrated in Figure 6. Of the Ch/poly(C-fu) films, Ch/poly(C-fu) (40/60) showed significant antibacterial activity against *E. coli* ATCC 43895 and *S. aureus* ATCC 6538, the zone of inhibition was determined to be 18 ± 0.5 mm and 18 ± 0.9 mm, respectively. The MIC of Ch/poly(C-fu) (40/60) against *S. aureus* and *E. coli* was determined to be 50 µg/mL. In addition, bactericidal efficacy of Ch/poly(C-fu) film increases with the increase in benzoxazine content (C-fu) up to 60% weight. Moreover, there is a decrease in antibacterial activity for the neat poly(C-fu). Surprisingly Ch/poly(C-fu) (80/20) film did not exhibit any antibacterial activity against the two bacterial strains. This clearly indicates that the synergistic effect of both Pbz and chitosan could be seen at a particular concentration [i.e., Ch/poly(C-fu) (40/60)], and above or below this concentration either reduced or no antibacterial activity was observed. We made similar observations in a previous study, in which we investigated the anti-corrosion and anti-biofouling properties of Pbz/copolymer coatings containing arbutin and silane [56]. The synthesized Ch/poly(C-fu) films possess anti-bacterial activity, as it contains both chitosan and curcumin. The poly cationic structure of chitosan is responsible for its anti-bacterial activity, as it binds to the negatively charged bacterial cell wall, resulting in disruption of cell and subsequently leading to cell death [57,58]. Whereas curcumin, by generating anti-oxidation products, inhibit bio-film formation and prevents bacterial growth [59,60]. Curcumin when combined with chitosan, as in Ch/poly(C-fu) films exerts a synergistic antibacterial effect. In case of Ch/poly(C-fu) (80/20 and 60/40), very less content of curcumin is present and in Ch/poly(C-fu) (20/80), the film has more cross-linked structure that hinders the availability of the active groups. Whereas, in Ch/poly(C-fu) (40/60), the structure has more active groups producing synergistic effect of both curcumin and chitosan.

### 3.7. Antibiofilm Potencies of Ch/poly(C-fu) (40/60) against E. coli and S. aureus

**Figure 7:**
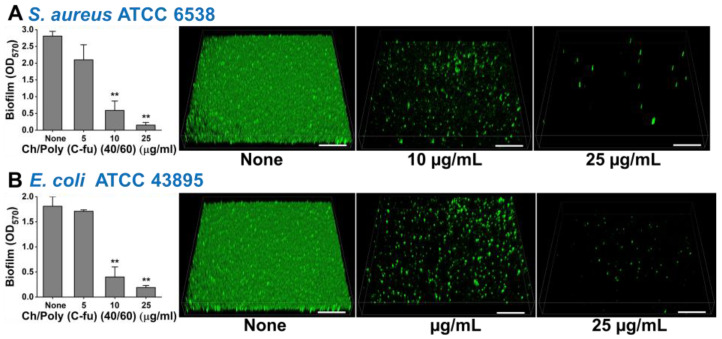
Effects of Ch/Poly (C-fu) (40/60) on *S. aureus*, and *E. coli* biofilm formation. Biofilm formations by *Staphylococcus aureus* ATCC 6538 (**A**), and Escherichia coli ATCC 43895 (**B**) quantified in the presence of Ch/Poly (C-fu) (40/60) after 24 h in 96-well plates. **, *p* < 0.01 vs. non-treated controls. Biofilm formation was observed by confocal laser microscopy. Scale bars represent 100 μm.

A biofilm assay was performed to examine the antibiofilm potency of Ch/Poly (C-fu) (40/60) *against E. coli* and *S. aureus*. Figure 7 shows the dose-dependent biofilm inhibitory effect of Ch/poly(C-fu) (40/60) at doses of 5, 10, or 25 µg/mL. As a result, Ch/poly(C-fu) (40/60) at 10 µg/mL inhibited 77.9 ± 11.0 and 79.0 ± 9.9% biofilm formation by *E. coli* and *S. aureus* after 24 h incubation, respectively. Furthermore, when the dose of Ch/poly (C-fu) (40/60) was increased to 25 µg/mL, biofilm inhibition increased to >89 ± 2.3 and 80 ± 0.3%, respectively.

**Figure 8:**
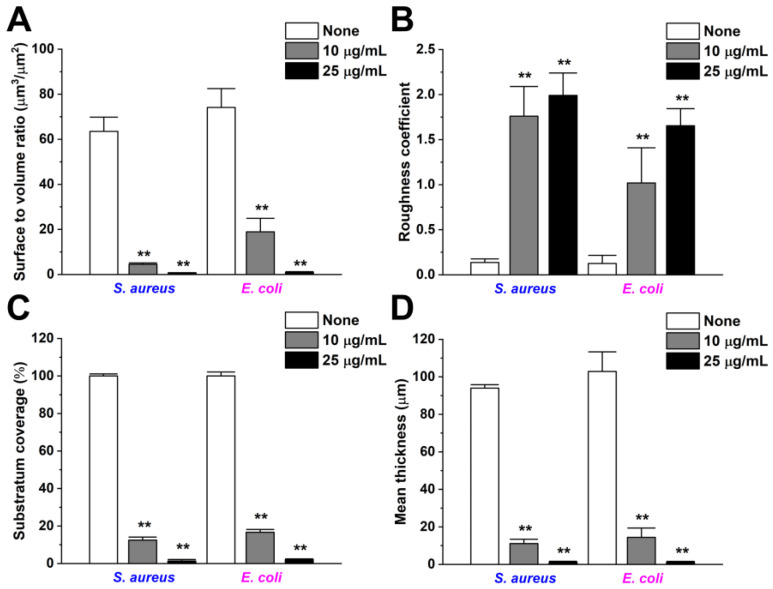
Effects of Ch/Poly (C-fu) (40/60) on *S. aureus*, and *E. coli* biofilm biomasses, surface to volume ratio (**A**), roughness coefficient (**B**), substratum coverages spatial characteristics (**C**) and mean thicknesses (**D**) were quantified by COMSTAT analysis. **, *p* < 0.01 vs. non-treated controls.

In the current study, the synthesized Ch/poly(C-fu) (40/60) inhibited selective *E. coli* and *S. aureus* biofilm, at lower concentration whereas with increased concentration, it showed potential antibacterial activity against both tested Gram-positive and Gram-negative bacterial strains. Additionally, biofilm reductions were confirmed by confocal laser microscopy (Figure 7) and COMSTAT biofilm analysis (Figure 8). More specifically, in 96-well plate both the pathogens *S. aureus*, and *E. coli* colonized the entire substratum very rapidly (substratum coverage 99–100% in 24 h). However, treatment with Ch/Poly (C-fu) (40/60) at 25 µg/mL reduced biomasses and mean thicknesses of *S. aureus* and *E. coli* biofilms by >95 and 97%, respectively versus untreated controls (Figure 8).

## 4. Conclusions

We developed a sustainable approach to graft curcumin and furfurylamine benzoxazine monomers on to chitosan at different ratios using a catalyst-free approach to produce Ch-graft-poly(C-fu). Polymeric structures contained physical and chemical crosslinks within Ch layers formed by ring-opening polymerization of C-fu. The gelatin behavior of chitosan is significantly reduced when C-fu is embedded; lower ratios resulted in self-scrolling, whereas higher ratios (60%) resulted in a self-supported film that was more mechanically stable. In addition to exhibiting its inherent functionalities, Ch galleries were expanded to expose phenolic and amine-rich substrates of C-fu origin, providing an enhanced crosslink density. Against the Gram-positive and Gram-negative pathogens—*S. aureus* and *E. coli*, Ch-graft-poly(C-fu) films showed significant antimicrobial activity. When *S. aureus* cells were treated with hybrid films, they were exposed to high levels of oxidative stress, leading to toxicity and cell death. Greener bio-based Ch-graft-poly(C-fu) hybrid polymers can be used to prevent wound-acquired infections in future health care systems. In addition, the reversibility of imine linkages in Ch with C-fu might confer and explain their improved antibacterial activity of Ch/C-fu films. Furthermore, based on green components the devised strategy enhanced the biocompatibility of films and improved tensile and antibacterial properties.

## Figures and Tables

**Table 1:** DSC data of poly(Ch/C-fu) blends.

Sample	T_Onset_ (°C)	T_Max_ (°C)	T_final_ (°C)	∆H (J/g)
Poly(Ch/C-fu) (0/100)	151	210	243	197
Poly(Ch/C-fu) (20/80)	144	204	237	152
Poly(Ch/C-fu) (40/60)	136	193	228	108
Poly(Ch/C-fu) (60/40)	130	190	223	63
Poly(Ch/C-fu) (80/20)	127	186	216	38
Poly(Ch/C-fu) (0/100)	-	-	-	-

**Table 2:** Antibacterial activity as indicated by growth-inhibition zone of poly (Ch/C-fu) at concentration of 200 µg/mL against *S. aureus* and *E. coli*.

Bacterial Strains	Inhibition Zone (mm)					
	CC 0/100	CC 20/80	CC 40/60	CC 60/40	CC 80/20	CC 100/0
*S. aureus* ATCC 6538	11 ± 1.1	9 ± 1.3	18.5 ± 0.9	9.5 ± 0.5	7 ± 0.5	11.5 ± 0.9
*E. coli* ATCC 43895	8 ± 2.3	10.5 ± 0.8	18 ± 0.5	10.5 ± 0.8	7 ± 0.7	12.7 ± 1.1

## Data Availability

Not applicable.
